# Dr. William M. Mercer

**Published:** 1903-09

**Authors:** 


### OBITUARY.

Dr. William M. Mercer, of Corunna, Ind., a graduate of the University of Buffalo in the class of 1872, died at his home, July 24, 1903 , aged 75 years.

